# The Clinical Utility of a Hand-Held Piezoelectric Scanner in the Detection of Early Tumor and Changes in Breast Texture

**DOI:** 10.7759/cureus.70586

**Published:** 2024-10-01

**Authors:** Joana Gonçalves, Francisco Nogueira, Frederico Stock, Filipe Martins, Isabel Fernandes, Rita Gameiro-dos-Santos, João Gramaça, Carolina Trabulo, Inês Ângelo, Idília Pina

**Affiliations:** 1 Medical Oncology, Unidade Local de Saúde do Arco Ribeirinho, Barreiro, PRT; 2 Biomedical Engineering, Glooma, Braga, PRT; 3 Medical Oncology, Unidade Local de Saúde do Tâmega e Sousa, Penafiel, PRT

**Keywords:** breast cancer, early detection, hand-held scanner, piezoelectricity, screening

## Abstract

Background

Breast cancer (BC) is one of the leading causes of cancer death in females worldwide. Screening with mammography (MMG) is limited in low- and middle-income countries (LMICs). The implementation of an affordable and effective screening method is crucial. The intelligent breast examination (iBE) has emerged as a portable device with a glove shape using piezoelectricity. This experimental study evaluates the effectiveness of the device by comparing it with mammography (MMG), breast magnetic resonance imaging (MRI), and clinical breast examination (CBE).

Methods

This study included patients admitted to the senology unit who were under surveillance in a medical oncology unit. iBE was performed after each CBE and compared with Breast Image Reporting and Data System (BI-RADS) classifications. MMG/MRI was classified as negative (BI-RADS ≤2) or positive (BI-RADS ≥3). Measures of accuracy and agreement between tests were calculated.

Results

A total of 103 females were included between September 2022 and September 2023, who underwent iBE, CBE, and MMG/MRI. CBE and MMG showed moderate agreement in categorization (ρ=0.99). With a specificity for predicting a negative MMG of 90.8% and a negative predictive value of 79.7%. Benign findings, cysts, fibroadenoma, and benign microcalcifications were presented in 80 patients (seromas and non-suspicious hypoechogenic images). The performance of iBE was evaluated by comparing the breast with alterations to the control breast within each BI-RADS categorization.

Conclusion

As of now, iBE does not identify breast changes. The improvement proposals emphasized the incorporation of accelerometer sensors, signal conditioning to allow for the collection of compression and decompression data from the sensors, and consideration of pressure stress. These improvements are crucial to optimize the iBE's ability to detect changes in breast texture, enhancing the iBE's effectiveness in the early detection of BC.

## Introduction

Breast cancer is globally the most common malignancy in females, and the most common cause of cancer death in the gender [1]. Early diagnosis and treatment evolution have reduced mortality by 50% in recent decades [2]. In high-income countries, mammography (MMG) is used for population-based screening. Nevertheless, MMG is expensive and resource-intensive, requiring an extensive infrastructure to link screening to additional diagnostic investigation. In addition, breast cancer in low- and middle-income countries (LMICs) is commonly present in females under the age of 45 years, for whom MMG is not recommended. Reducing the cost of screening methods can contribute to early diagnosis of breast cancer, increasing the probability of successful treatment [3-5].

Studies on the implementation of clinical breast examination (CBE) in LMICs have shown efficacy in screening and detection of breast cancer, but failure in the follow-up, as well as the potential to increase false positive rates and elevate anxiety levels among patients [6,7]. A method to standardize the different breast tissue densities observed in CBE and correlate them with an imaging report is needed, which would make the link to this additional screening/diagnostic method more reliable. Piezoelectric palpation (intelligent breast examination {iBE}) can improve patient screening. It is a portable and low-cost device, with a short learning curve and provides electronic documentation for diagnostic research [8-15]. The device generates an electrical voltage when it is physically deformed, i.e., when it detects a physical change in the form of a vibration, sound wave, or mechanical stress, and can detect clinically significant breast lesions with a sensitivity of 87% [16]. Furthermore, current evidence supports the implementation of the device as a complement to CBE and MMG for breast screening in countries with limited resources [17,18]. As the iBE device continues to develop, it is important to summarize current technologies, benchmark its existing performance, and identify areas for improvement [19]. This experimental study aimed to compare the iBE's performance in detecting changes in breast texture in a cohort of patients with MMG or MRI performed within ≤6 months.

The abstract of this article was previously presented at the European Society for Medical Oncology (ESMO) Breast Cancer Congress 2024.

## Materials and methods

Study design

This is an observational study with no group control designed to assess the applicability of iBE in identifying changes in breast tissue texture, whether malignant or benign. This study was approved by the Ethics Committee of Unidade Local de Saúde do Arco Ribeirinho (ULSAR) and all patients signed an informed consent form prior to their participation.

**Figure 1:**
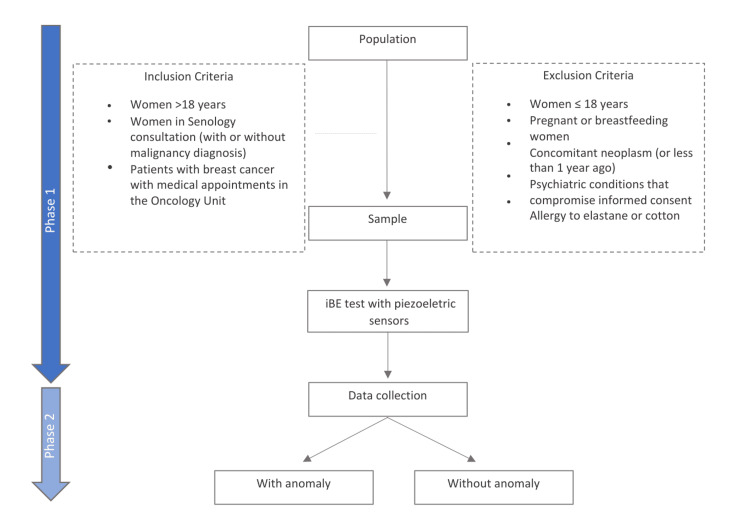
Study design and inclusion and exclusion criteria. iBE: intelligent breast examination

From September 2022 to September 2023, we included adult female patients (>18 years of age) from the senology consultation (with or without a malignancy diagnosis) and patients from the medical oncology unit. The exclusion criteria were patients under the age of 18 years, pregnant or breastfeeding females, patients with a second concomitant neoplasm or neoplasm in the previous year, patients with psychiatric conditions compromising informed consent, and patients allergic to elastane or cotton (Figure 1).

**Figure 2:**
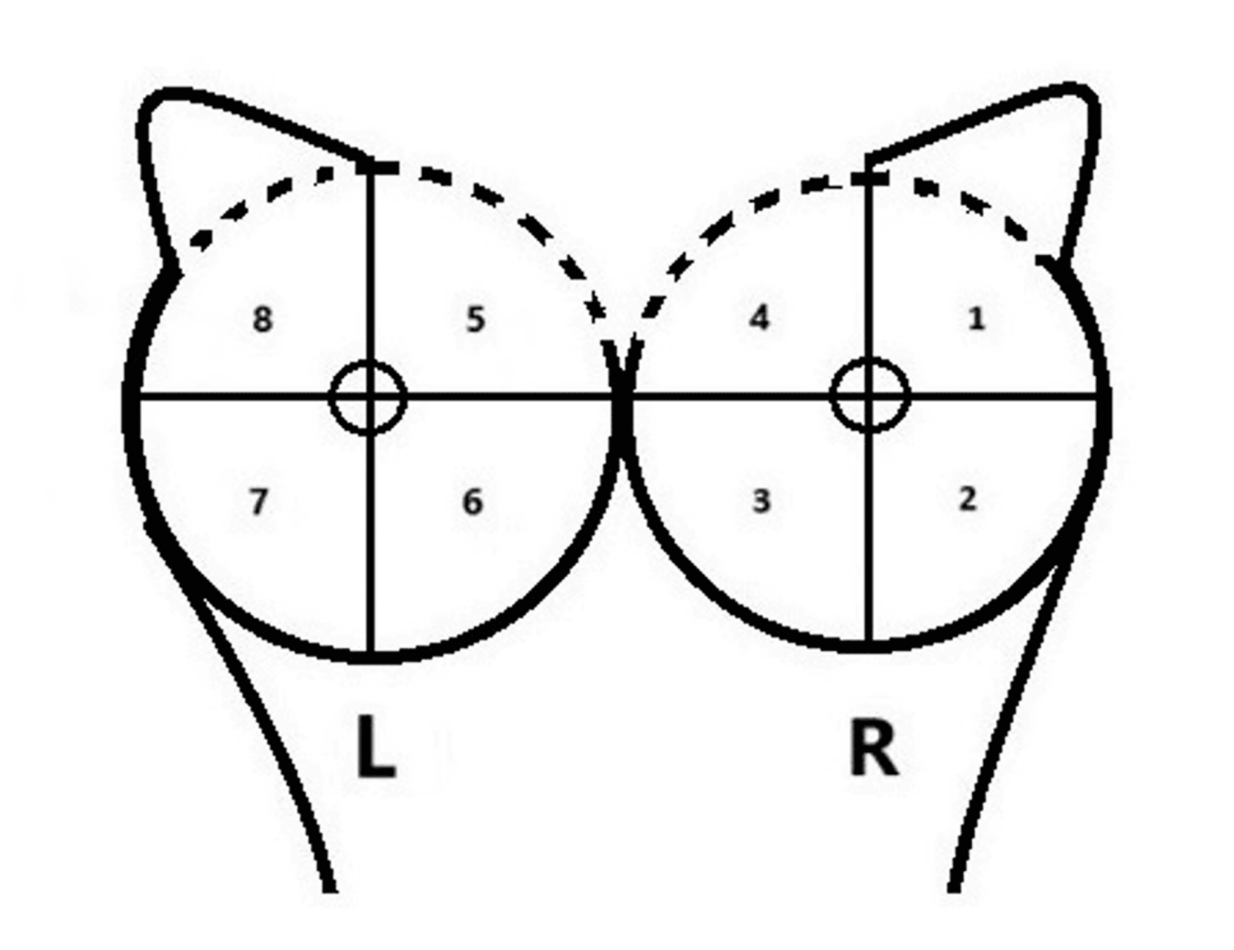
Pathway between different breast quadrants. L: left breast; R: right breast; 1: upper external quadrant; 2: interior external quadrant; 3: Inferior internal quadrant; 4: upper internal quadrant; 5: upper internal quadrant; 6: inferior internal quadrant; 7: inferior external quadrant; 8: upper external quadrant

CBE was performed in a supine position prior to the bilateral iBE, following this order for the left breast: upper external quadrant (UEQ), inferior external quadrant (IEQ), inferior internal quadrant (IIQ), upper internal quadrant (UIQ); and for the right breast: inferior internal quadrant (IIQ), upper internal quadrant (UIQ), upper external quadrant (UEQ), and bilateral axillary palpation (Figure 2).

The numerical data from the electrical voltage generated by the iBE scan using computer analysis were correlated with the results categorized according to the Breast Image Reporting and Data System (BI-RADS) classification of the MMG and/or MRI, performed in a time interval of ≤6 months. BI-RADS 1 or 2 classification was defined as a negative examination and BI-RADS ≥3 was defined as positive. Only data from the breast parenchyma were used to calculate the correlation between variables, excluding iBE from the axilla. After computer analysis and data standardization, the results of the iBE scan were correlated with those of MMG and/or MRI, CBE, and the anatomopathological results of patients who underwent biopsy (BI-RADS ≥4).

iBE device

The iBE is a prototype of a medical device in the shape of a glove (92% cotton, 8% spandex), with eight piezoelectric sensors that sit on the fingers of the glove. The medical device (MD) also consists of the following components: Arduino Nano, a prototyping board containing an 8-bit ATMega-328P microcontroller connected to the eight sensors; a 9V battery, the power source for this MD; a Bluetooth module connected to the prototyping board for data transmission and a tablet.

The iBE communicates wirelessly with the tablet to display results in real-time, store and share data digitally, and compare results with previous results. The piezoelectric finger technology consists of tactile pressure sensors that measure tissue compression through electrical displacement when tactile palpations are made from top to bottom against the surface of the breast, specifically between 0 and 5V. The Arduino converts this voltage into numerical values for a range between 0 and 1023, where 0V corresponds to the value 0 and 5V to the value 1023. The iBE results are displayed under the numerical formula (unit=bits) on the tablet. Sensors with 20 mm were tested, with a resonance frequency (Fs)=2.0 kHz±0.5 and a capacitance (Co)=70 nF±30%. The medical device (iBE) received approval from Portugal’s regulatory agency for pre-market clinical trials involving intervention for the detection of breast findings.

Data collection and classification

The patient's age and the results of the iBE and CBE were recorded in a database at the time they were carried out. Test results (MMG or MRI) were accessed via the medical record or electronic medical record. The results of the CBE, iBE, and imaging tests were classified as negative or positive for comparative statistical analysis. The absence of a palpable lesion on CBE was classified as negative, while the presence of any clinically palpable lesion on CBE was classified as a positive examination. All patients with the inclusion criteria underwent iBE, even those whose CBE was negative. Benign findings on MMG (BI-RADS 1 and 2) were classified as negative, and suspicious microcalcifications, asymmetries or suspicious masses on MMG (BI-RADS 3, 4, or 5) were classified as a positive examination. Patients with a BI-RADS 6 breast examination were also classified as a positive examination.

Statistical analysis

An exploratory analysis was performed on the data obtained from the iBE glove, which was later confirmed with electronic schematic diagrams. An analysis was carried out in terms of frequency/amplitude (Fourier Transform), principal component analysis (PCA), and statistical analysis of the evolution of the signal in a specific time window, which was correlated with the original diagnosis.

Regarding diagnostic accuracy, measures including sensitivity, specificity, and Spearman's correlation coefficient (ρ) were calculated between the CBE (predicted) and MMG/MRI (actual/real) variables. The accuracy, sensitivity, and specificity were calculated directly using the confusion matrix values. The analysis of the comparison of readings (iBE) between the breast with alterations and the respective control breast of each category (BI-RADS ≥3) was carried out using the confusion matrix and Spearman's.

## Results

In total, 103 patients were included in the study with a median age of 61 (30-91) years. Forty-eight patients were excluded for missing values due to damage to the piezoelectric sensors. There were 29 positive CBE results and 38 positive MMG/MRI - BI-RADS ≥3 (Table 1). In this last group, nine patients already had a confirmed positive anatomopathological diagnosis (BI-RADS 6) and 16 patients underwent biopsy for additional diagnostic tests (BI-RADS 4 and 5).

**Table 1:** Characteristics of the study participants. CBE: clinical breast examination; MMG: mammography; BI-RADS: Breast Image Reporting and Data System

Characteristics	n=103
Mean age, years (range)	61 (30-91)
Race	
White, n	99
African origin, n	4
Negative examination detection	
CBE, n	74
MMG/MRI	65
BI-RADS 1, n	2
BI-RADS 2, n	63
Positive examination detection	
CBE, n	29
MMG/MRI, n	38
BI-RADS 3, n	13
BI-RADS 4, n	7
BI-RADS 5, n	9
BI-RADS 6, n	9

Negative results were found in 59 patients on both CBE and MMG, producing a specificity for predicting a negative mammogram of 90.8% (Table 2). This corresponds to a false positive rate of 9.23% and a negative predictive value of 79.7%. MMG/MRI and CBE showed significant agreement in categorization (ρ=0.99). There were 23 patients with a positive result on both CBE and breast examinations (MMG/MRI), producing a sensitivity for predicting a positive mammogram (BI-RAD 3, 4, 5, or 6) of 60.53%.

**Table 2:** CBE vs. MMG/MRI for positive examination detection. CBE: clinical breast examination; iBE: intelligent breast examination; MMG: mammogram; NPV: negative predictive value; Sp: specificity

Value	MMG/MRI (+)	Mammogram/MRI (-)	Measure
CBE (+)	23	6	-
CBE (-)	15	59	NPV=79.7%
Measure	-	Sp=90.8%	ρ=0.99

Benign findings, cysts, fibroadenoma, and benign microcalcifications were presented in 80 patients (seromas and non-suspicious hypoechogenic images). Two patients had no findings (two mastectomies). An initial diagnosis of undefined mass was given to two patients, corresponding to BI-RADS 4a and BI-RADS 4b. Both underwent a biopsy, revealing granulomatous mastitis and sclerosing adenosis with associated apocrine metaplasia. Of the 19 patients with a final diagnosis of invasive ductal carcinoma of the breast or ductal carcinoma in situ (DCIS), 11 had a BI-RADS 6 examination, nine had a BI-RADS 5 examination, and one had a BI-RADS 4 examination. Two patients had bilateral invasive breast carcinoma (BI-RADS 5). All malignant lesions were detected by breast examinations - MMG or MRI (Table 3).

**Table 3:** List of breast examination findings. MMG: mammography; CBE: clinical breast examination; DCIS: ductal carcinoma in situ

Variables	Number	MMG/MRI (+)	CBE (+)
No. findings	2	0	0
Benign findings	63	10	8
Cyst	3	2	1
Fibroadenoma	6	4	2
Microcalcifications	8	1	1
Mass	2	2	1
DCIS or breast cancer	19	19	16

iBE data from BI-RADS 6 (n=9), BI-RADS 5 (n=9), and BI-RADS 4 (n=7) were recorded and compared respectively with the control breast. Abnormalities were found in the right breast in seven patients (BI-RADS 6), six patients (BI-RADS 5), and three patients (BI-RADS 4). With regard to the pathologies found, eight patients with BI-RADS 6 had invasive ductal carcinoma, and one patient had DCIS. Nine patients with BI-RADS 5 had invasive ductal carcinoma, two of whom had bilateral invasive ductal carcinoma. One patient with BI-RADS 4 had invasive ductal carcinoma, and six patients had benign findings. With regard to the size of the neoplasms found, the median (2 cm) was very similar, except for the BI-RADS 4 neoplasm with a size of 0.8 cm. With regard to breast tumor location, there were no significant discrepancies (Table 4).

**Table 4:** General characteristics of breast tumor. LB: left breast; RB: right breast; CDIS: carcinoma ductal in situ; CBE: clinical breast examination; UEQ: upper external quadrant; IEQ: inferior external quadrant; IIQ: inferior internal quadrant; UIQ: upper internal quadrant; BI-RADS: Breast Image Reporting and Data System; iBE: intelligent breast examination

Variables	BI-RADS 6		BI-RADS 5		BI-RADS 4	
	Affected breast, n=9	Control, n=9	Affected breast, n=11	Control, n=7	Affected breast, n=7	Control, n=7
Breast						
LB	7	2	6	3	3	4
RB	2	7	5	4	4	3
Pathology						
Invasive ductal carcinoma	8	0	11	0	1	0
CDIS	1	0	0	0	0	0
Bening findings	0	0	0	0	6	0
Size (cm)						
Median	2.1	-	2	-	0.8	-
Minimum	1.1	-	0.7	-	0.1	-
Maximum	8	-	3.2	-	2.5	-
Location						
UEQ	3	-	4	-	3	-
IEQ	2	-	0	-	0	-
IIQ	2	-	2	-	1	-
UIQ	1	-	5	-	2	-
Nipple	0	-	0	-	1	-
More than one	1	-	0	-	0	-
iBE result (bits)						
Minimum	0	0	0	0	0	0
Maximum	15-166	13-142	10-60	15-71	17-140	15-159
CBE						
Positive	8	0	9	0	2	0
Negative	1	9	2	7	5	7
Missing	0	0	0	0	0	0
MMG/MRI result						
Positive	9	0	11	0	7	1
Negative	0	9	0	7	0	6

**Figure 3:**
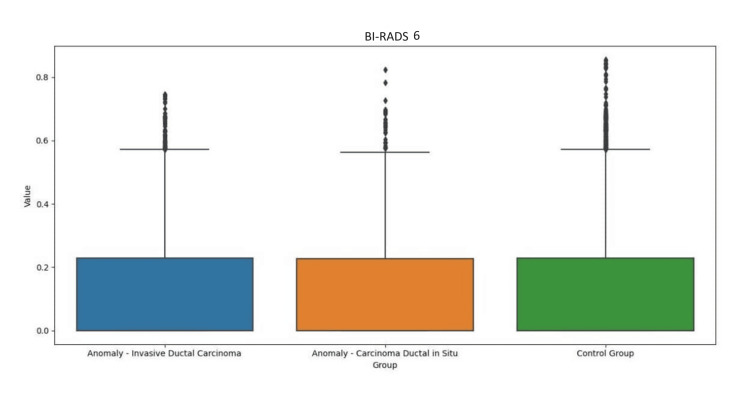
Comparison of iBE performance between breast with anomaly (BI-RADS 6) and control breast. BI-RADS: Breast Image Reporting and Data System; iBE: intelligent breast examination

**Figure 4:**
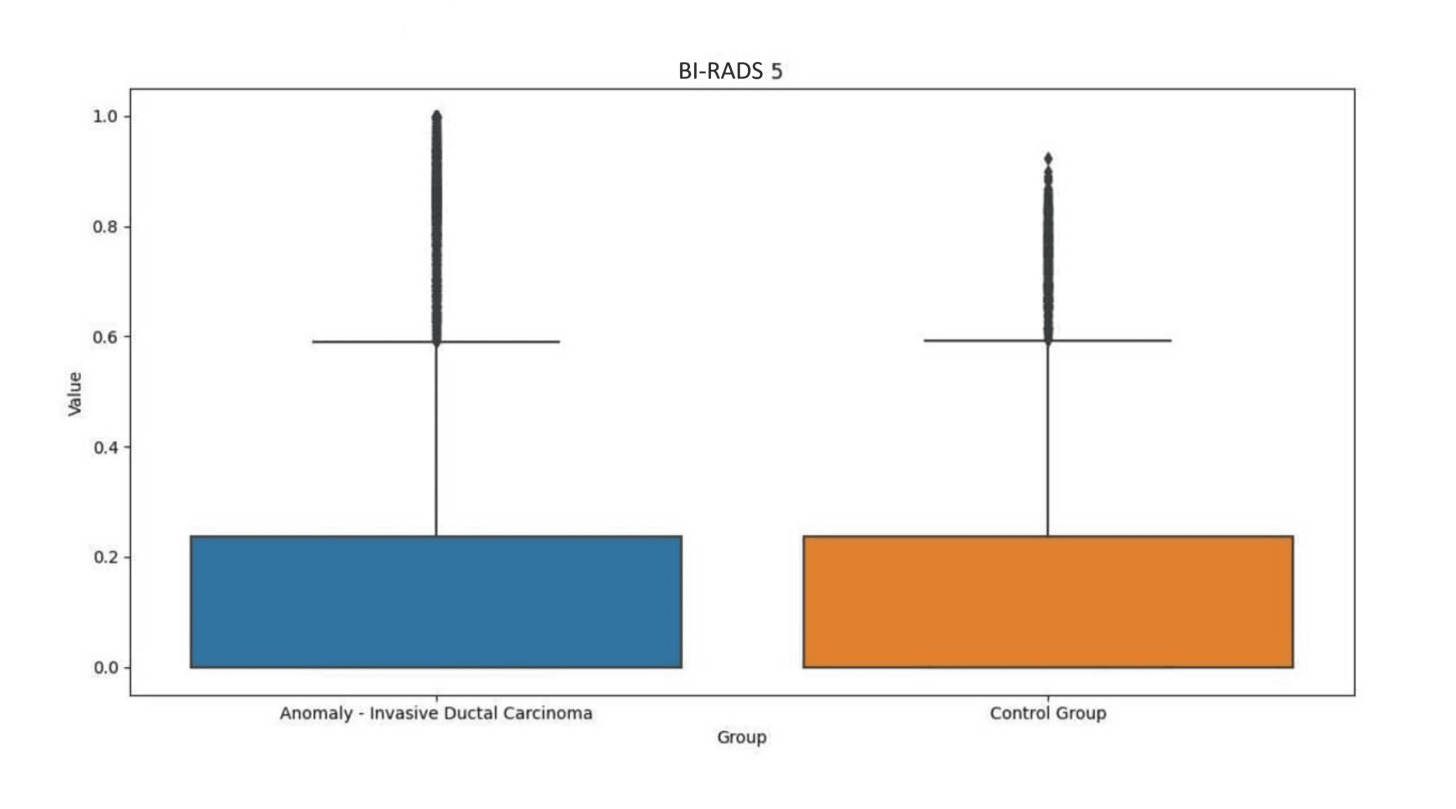
Comparison of iBE performance between breast with anomaly (BI-RADS 5) and control breast. BI-RADS: Breast Image Reporting and Data System; iBE: intelligent breast examination

**Figure 5:**
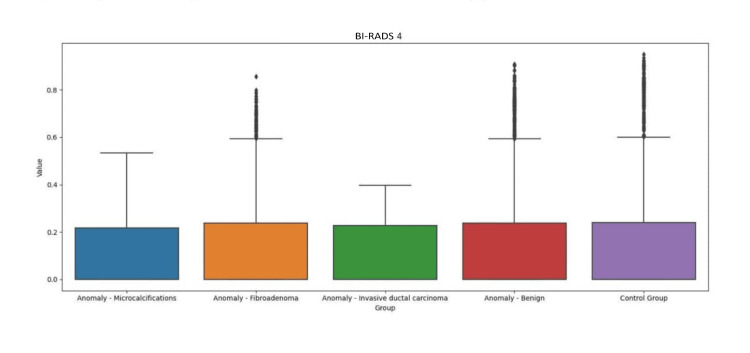
Comparison of iBE performance between breast with anomaly (BI-RADS 4) and control. BI-RADS: Breast Image Reporting and Data System; iBE: intelligent breast examination

Regarding the results of iBE performance, we describe the minimum and maximum values (bits) associated with breasts with abnormalities (15-166 bits BI-RADS 6; 10-60 bits BI-RADS 5; 17-140 bits BI-RADS 4) vs. control breast (13-142 bits BI-RADS 6; 15-71 bits BI-RADS 5; 15-159 bits BI-RADS 4). There was no numerical translation that allowed us to gauge sensitivity and/or specificity with regard to the performance of iBE in the detection of changes in breast texture. There was no correlation between iBE and MMG to date (Figures 3-5).

In patients with BI-RADS 1, the average duration was approximately 39.1 seconds with a median of 41.3 seconds. For BI-RADS 2-3, the average duration was around 42.9 seconds with a median of 44.1 seconds. For BI-RADS 4-6, the average duration was 55.7 seconds with a median of 53.6 seconds. With regard to CBE, all the BI-RADS 6 neoplasms were detected, 80% (n=7) of the BI-RADS 5 neoplasms while the undetected tumors were around 11 mm and 7 mm. Within the BI-RADS 4 categorization, the nodule corresponding to invasive ductal carcinoma was detected. The other nodule BI-RADS 4 was identified measuring around 22x25 mm and corresponding to a canalicular fibroadenoma. With regard to the MMG/MRI results, the respective imaging examination showed a sensitivity of 100% in detecting breast neoplasia. When assigning the BI-RADS 4 category, the imaging examination overestimated the benign findings detected. A BI-RADS 4 in a control breast corresponded to a cyst.

## Discussion

Many countries lack infrastructures for breast cancer screening and are in need of a low-cost and low-risk method for females not eligible for the gold standard MMG. Even in countries with well-established screening procedures, a fast, painless, and cost-effective method would be valuable.

In this context, the iBE prototype emerges as an inexpensive, portable, and independent tool from radiology, aimed at identifying females in need of complementary evaluation. This study intended to validate this innovative device, potentially capable of early breast cancer detection and to become a possible screening method, eventually influencing survival outcomes and quality of life (QoL).

Clinical limitations

Clinically, the use of the iBE device did not compromise the medical consultation due to its simple and intuitive design and portability. Its simplicity could offer an efficient way of carrying out regular examinations and could potentially revolutionize healthcare logistics by making examinations more accessible and convenient, increasing the chances of early diagnosis. However, some limitations were observed as below.

Study Design

The study was conducted by a single investigator in a consultation setting, which reduces variability and representativeness. Moreover, the investigator had prior access to the patients' BI-RADS classifications, potentially introducing bias, as pre-existing knowledge could influence the assessment process subconsciously, limiting the objectivity of breast abnormality detection.

Bias in Examination Time and Force

It was noted that the pressure applied by the investigator and the time spent on each analysis varied, which influenced the examination results. For example, cases with BI-RADS ≥4 showed a tendency toward longer examination times, potentially reflecting unconscious bias related to the known condition of the patient.

Proposed Solutions for Clinical Limitations

To address these clinical limitations, we propose that future studies involve multiple clinical professionals applying the device without prior knowledge of the patients' mammography results. This will minimize bias and enhance the reliability and objectivity of the findings. Additionally, standardizing the force applied and the time dedicated to each analysis would improve consistency.

Technical and design limitations

The early prototype revealed several limitations related to the design and electronics, which compromised the adequacy of the data. The limitations are explained below.

Sensor Signal Issues

The device suffered from under-sampling, with an insufficient sampling rate to adequately capture the signal variation, resulting in an incomplete representation of the original signal. Furthermore, the signals generated by the sensors have alternating current (AC) characteristics around 0 volts, but the analog-to-digital conversion circuitry could only process signals ≥0 volts. Consequently, essential information regarding compression was lost, retaining only the decompression data.

Mechanical Stress and Failures

Mechanical stress caused failures in the device’s hardware components, highlighting issues in the design that affect its durability and ease of use.

Absence of Inertial Measurement Unit (IMU)

The lack of an IMU prevented the determination of the glove's position relative to the breast, which is crucial for inferring not only the size of anomalies but also the affected area and for tracking the evolution of nodules over time. This significantly impacted the device’s clinical potential.

Statistical limitations and data analysis

Statistical analyses were performed, generating multiple features (e.g., max. value, min. value, trend, percentile, mean, median, peak count, and standard deviation) from the multivariate time series data collected during the examinations. These features were calculated both for the entire examination and in a sliding window with n samples. However, the results from classifiers like feedforward neural networks and random forests indicated an inability to detect anomalies accurately, as reflected by confusion matrices (Table 5).

**Table 5:** Confusion matrix resulting from the classifier. 0: healthy breast (BI-RADS ≤1, 2); 1: unhealthy breast (BI-RADS >2); 00: both breasts healthy; 01: right breast unhealthy; 10: left unhealthy; 11: both with abnormalities. MMG: mammography; BI-RADS: Breast Image Reporting and Data System; iBE: intelligent breast examination

Actual values (MRI/MMG)	Predicted values (iBE)			
	00	01	10	11
11	4	0	0	0
10	2	0	0	1
01	3	0	0	0
00	4	0	0	0

Proposed Statistical Enhancements

For future iterations, improving the statistical models, increasing the sampling rate, and refining the signal processing methods are crucial steps. Using normalized data and a sliding window approach could further enhance classification performance, leading to more accurate anomaly detection.

## Conclusions

While the iBE prototype presents clinical, technical, and statistical limitations, this study has provided valuable insights for the next phase of development. Improvements in signal processing, incorporation of an IMU, and reduction of examiner bias will be essential steps moving forward. These enhancements will pave the way for a multicenter trial, aiming to establish the iBE glove as a reliable prescreening tool for breast cancer.
