# Correction: The Clinical Utility of a Hand-Held Piezoelectric Scanner in the Detection of Early Tumor and Changes in Breast Texture

**DOI:** 10.7759/cureus.c293

**Published:** 2025-09-16

**Authors:** Joana Gonçalves, Francisco Nogueira, Frederico Stock, Filipe Martins, Isabel Fernandes, Rita Gameiro-dos-Santos, João Gramaça, Carolina Trabulo, Inês Ângelo, Idília Pina

**Affiliations:** 1 Medical Oncology, Unidade Local de Saúde do Arco Ribeirinho, Barreiro, PRT; 2 Biomedical Engineering, Glooma, Braga, PRT; 3 Medical Oncology, Unidade Local de Saúde do Tâmega e Sousa, Penafiel, PRT

The Ethics Statement and Conflict of Interest Disclosures section has been updated to disclose the following:

**Financial relationships:** Francisco Nogueira, Frederico Stock and Filipe Martins declare(s) employment and stock/stock options from CleoCare (formerly known as Glooma). CleoCare is a biomedical engineering company which produces and sells an iBE medical device, the SenseGlove. 

